# Supplementary figures and images for: The Zebrafish Meiotic Cohesin Complex Protein Smc1b Is Required for Key Events in Meiotic Prophase I
Source: Front Cell Dev Biol. 2021 Aug 9;9:714245. doi: 10.3389/fcell.2021.714245 (PMC8381726; doi:10.3389/fcell.2021.714245)

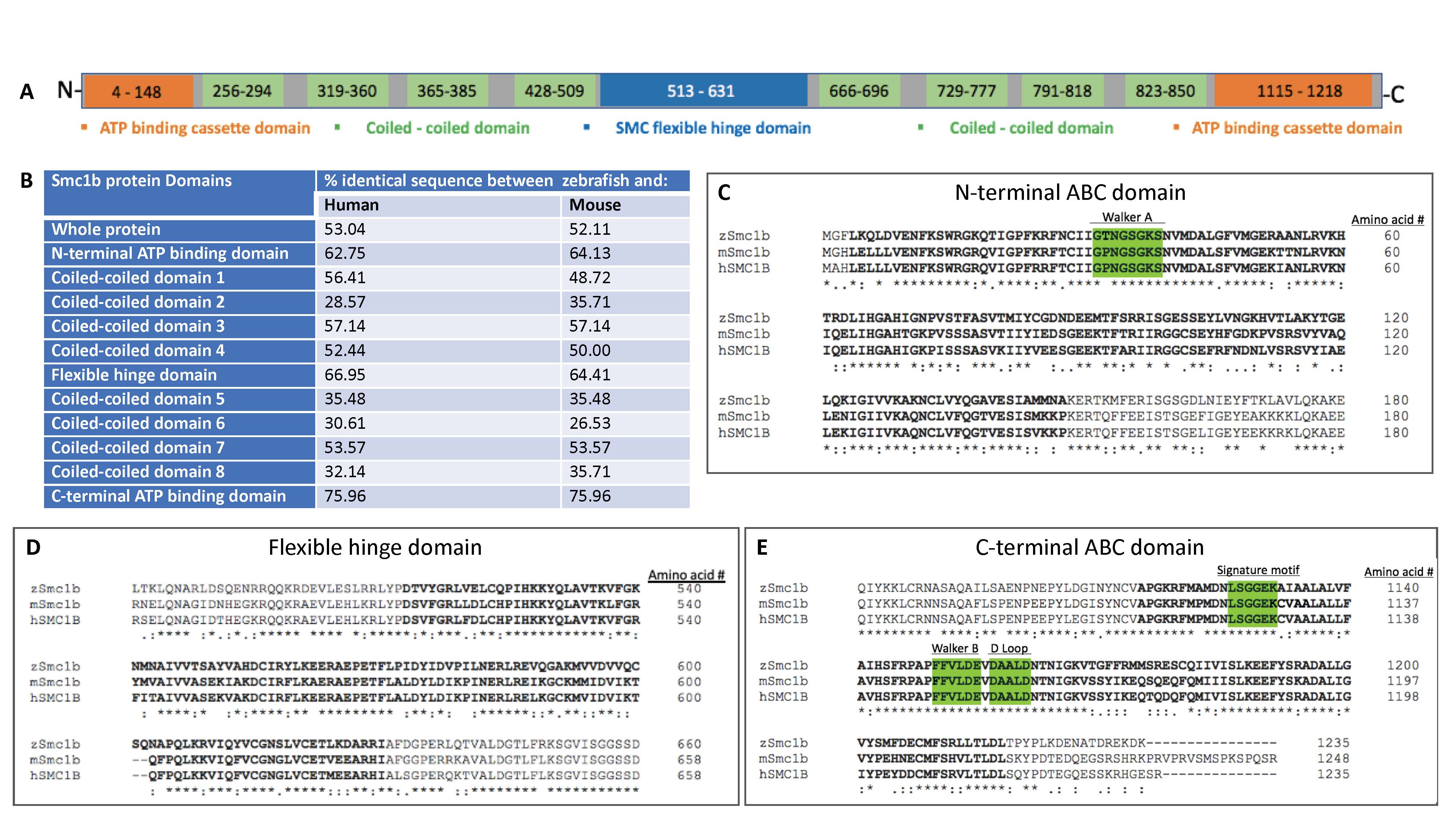

Supplement: Supplementary Figure 1 — Conservation of key motifs in vertebrate Smc1b proteins. (A) Graphical representation of zebrafish Smc1b protein. Numbers inside boxes represent amino acid position in the corresponding domain. (B) Percentage of identical amino acid residues between zebrafish (z) and either human (h) or mouse (m) Smc1b proteins. (C–E) Protein sequence alignments of the N-terminal ATP binding domain (C), flexible hinge domain (D), and C-terminal ATP binding domain (E). The conserved motifs are highlighted in green. The consensus motif sequences are Walker A: GxxGxGK (S/T), ABC transporter signature motif: LSGG(E/Q) (K/R), Walker B: hhhhDE, where x is any of 20 amino acid and h is any hydrophobic residue. [file Image_1.JPEG]
